# Construction of a genetic linkage map in *Pyropia yezoensis* (Bangiales, Rhodophyta) and QTL analysis of several economic traits of blades

**DOI:** 10.1371/journal.pone.0209128

**Published:** 2019-03-08

**Authors:** Linbin Huang, Xinghong Yan

**Affiliations:** 1 Key Laboratory of Exploration and Utilization of Aquatic Genetic Resources (Shanghai Ocean University), Ministry of Education, Shanghai, P. R. China; 2 National Demonstration Center for Experimental Fisheries Science Education (Shanghai Ocean University), Shanghai, P. R. China; 3 International Research Center for Marine Biosciences at Shanghai Ocean University, Ministry of Science and Technology, Shanghai, P. R. China; BIG-CEA Grenoble, FRANCE

## Abstract

*Pyropia yezoensis* is an economically important seaweed but its molecular genetics is poorly understood. In the present study, we used a doubled haploid (DH) population that was established in our previous work to construct a genetic linkage map of *P*. *yezoensis* and analyze the quantitative trait loci (QTLs) of blades. The DH population was genotyped with fluorescent sequence-related amplified polymorphism (SRAP) markers. A chi-square test identified 301 loci with normal segregation (*P* ≥ 0.01) and 96 loci (24.18%) with low-level skewed segregation (0.001 ≤ *P* < 0.01). The genetic map was constructed after a total of 92 loci were assembled into three linkage groups (LGs). The map spanned 557.36 cM covering 93.71% of the estimated genome, with a mean interlocus space of 6.23 cM. Kolmogorov-Smirnov test (α = 5%) showed a uniform distribution of the markers along each LG. On the genetic map, 10 QTLs associated with five economic traits of blades were detected. One QTL was for length, one for width, two for fresh weight, two for specific growth rate of length and four for specific growth rate of fresh weight. These QTLs could explain 2.29–7.87% of the trait variations, indicating that their effects were all minor. The results may serve as a framework for future marker-assisted breeding in *P*. *yezoensis*.

## Introduction

*Pyropia yezoensis* is a marine red alga with high nutritional values and is one of the most important maricultural crops across the world, mainly in Japan, Korea and China [1]. During the cultivation of *P*. *yezoensis*, hundreds of tons of nutrients (nitrogen and phosphorus) are removed from the eutrophic seawater by blade harvest every year [2]. However, some problems such as germplasm degeneration, frequent diseases and bad harvests [3–5] have arisen under the influence of global warming [6]. Therefore, new varieties with higher yield, stronger resistance to abiotic stress and greater ecological adaptability are urgently needed for sustained development of *Pyropia* industry.

The traditional breeding methods of *P*. *yezoensis* are based on either observed variations by selecting blades with induced variants [3, 7, 8], or controlled crosses by selecting blades presenting recombination of desired genes from different parents [9–11]. However, traditional breeding is usually time-consuming and inefficient [12], and has a limited ability to breed complex characters [13]. Fortunately, progress in molecular genetics has enabled plant breeders to directly select genotypes, thereby accelerating crop improvement [14], and molecular marker-assisted selection (MAS) has become the main direction of plant breeding [15–17]. For MAS, construction of a genetic linkage map is one of the most important steps [18]. To date, genetic linkage maps have been successively constructed in dozens of different species of plants and animals and have played important roles in various studies [19]. However, genetic linkage map construction in seaweeds remains in its infancy and has been reported only in five important species, including *Laminaria japonica* [20], *L*. *longissima* [21], *Ectocarpus siliculosus* [22], *Porphyra haitanensis* [23] and *Undaria pinnatifida* [24]. Those maps have been used for quantitative trait locus (QTL) detection of economic traits [25, 26], mapping of sex-linked loci [24, 27] and large-scale assembly of genome sequence [22]. The reasons for the lag of seaweed maps include that the biological properties of seaweed at the molecular level is poorly understood [28], polymorphic molecular markers such as the most commonly used SSR markers are lacking [21, 23], and establishing a mapping population using highly heterozygous parents is difficult [20]. For *P*. *yezoensis*, the blades are monoecious and could be self-fertilized, and the heterozygote could only be identified by F_1_ blades if they are mainly color-sectored [29], which depended on tissue culture techniques. Except the doubled haploid (DH) population established in our previous work [30], there is no other mapping population of *P*. *yezoensis* reported. This might be the main reason why no genetic linkage map for *P*. *yezoensis* was constructed.

The economically important traits of gametophytic blades in *P*. *yezoensis* are quantitatively inherited traits controlled by multiple genes [30]. By means of genetic mapping, quantitative traits can be decomposed into multiple QTLs, and the genetic basis of complex quantitative traits can be clarified [31]. In the present study, a genetic linkage map of *P*. *yezoensis* was constructed using sequence-related amplified polymorphism (SRAP) markers and high-performance capillary electrophoresis analysis based on a DH population, for further QTL detection of economic traits of gametophytic blades. Our findings will facilitate the future application of MAS in breeding varieties in *P*. *yezoensis*.

## Materials and methods

### Plant materials

Two parental strains of *P*. *yezoensis* with different economic traits were used in this study. Py-HT was a red-type pigmentation mutant whose gametophytic blade was fast growing, thin, contained high content of major photosynthetic pigments and was resistant to high-temperature. Py-LS was a wild-type strain whose gametophytic blade was slow growing, thick, contained low content of major photosynthetic pigments, and was poorly resistant to high-temperature [32]. In our previous work, Py-HT and Py-LS were used as maternal and paternal parent, respectively, in an intraspecific cross because they are monoecious and could be self-fertilized. The heterozygote (heterozygous conchocelis) was identified according to the method described in Yan and Aruga (29), for the construction of a DH mapping population [30], which was used in the present work. Briefly, only four-color sectored mosaic blades were screened from the F_1_ blades that developed from the conchospores released from the heterozygous conchocelis. Every selected blade was then cut into four color-sectors along the boundaries of adjacent color-sectors and every color-sector was subsequently cultured individually. A DH strain was obtained when one of the carpospores was released from a self-fertilized color-sector and developed into a single conchocelis. Finally, a mapping population containing 148 DH strains was established from 37 four-color sectored mosaic blades (http://dx.doi.org/10.17504/protocols.io.x3vfqn6). All strains were conserved in our laboratory in the form of free-living conchocelis at 19±1°C under a photon flux density of 10±1 μmol photons m^-2^ s^-2^ (10:14 LD) provided by cool-white, 40-W fluorescent lamps according to the method described by Kato and Aruga [33].

### DNA extraction

Genomic DNA was isolated from 30–40 mg (fresh weight) of free-living conchocelis of each DH and parent using a Plant Genomic DNA Kit (DP305, TIANGEN) with modified sample treatment. Briefly, the conchocelis was sucked dry of culture solution and cut into a smooth paste in 100 μL deionized water with a single edge razor blade. DNA was extracted from the sample according to the manufacturer’s protocol. The concentration and purity of DNA were determined based on the spectrophotometric absorbance and the ratio of OD260/OD280 (Nanodrop 2000, Thermo Fisher Scientific). The size and integrity of DNA were assessed by 1.0% agarose gel electrophoresis. DNA with high quality was diluted to 30 ng ∙ μL^-1^ with Tris-EDTA buffer solution and stored at -20°C for further experiments.

### Polymorphic primers screening

The sequences of 21 forward primers and 21 reverse primers (Table 1) were obtained from original papers [34–36] and designed according to the method described in Li and Quiros [37]. After random pairing, 441 primer combinations were obtained. Primers were synthesized in Sangon Biotech (Shanghai) Co., Ltd (Shanghai, China) and amplified in two parents and four DH strains to screen primer combinations with rich polymorphic loci. The PCR reaction was carried out in 15.0 μL solution containing 7.5 μL Taq PCR Master Mix (B639293, Sangon Biotech), 1.0 μL forward and 1.0 μL reverse primers (20.0 μM), 1.0 μL genomic DNA (30.0 ng ∙ μL^-1^) and 4.5 μL deionized water. The SRAP procedure was performed as previously described in Li and Quiros [37]. PCR products were separated by electrophoresis on an 8% non-denaturing polyacrylamide gel [Acryl/Bis (29:1), 1×TBE] (native-PAGE) and photographed with Gel Imaging System (Gel Doc XR+, Bio-Rad) after rapid and economic silver staining [38]. Briefly, the gel was washed twice with deionized water for 60 s every time (the same below) and stained with 300 mL silver nitrate solution (0.1% w/v) for 15–20 min. Then, the gel was washed twice and developed in 300 mL sodium hydroxide solution (1.6% w/v, including 300 μL formalin) until the bands were clear with a blemish-free background. Finally, the gel was washed twice and photographed. Bands were detected and analyzed using Image Lab Software (version 5.1, Bio-Rad) according to the instruction manual. Primer combinations were screened for those that amplified abundant bands. To save cost, only those primers with high frequencies among the combinations were labeled with 5’-HEX (Hexachloro fluorescein phosphoramidite) and paired with ordinary primers for further genotyping.

**Table 1:** Sequence-related amplified polymorphism primers used to detect DNA polymorphisms among parents and doubled haploid population of *Pyropia yezoensis*.

**Forward primers and sequence (5’-3’)**		
Me1: TGAGTCCAAACCGGATA	Me2: TGAGTCCAAACCGGAGC	Me3: TGAGTCCAAACCGGATG
Me4: TGAGTCCAAACCGGACC	Me5: TGAGTCCAAACCGGGAT	Me6: TGAGTCCAAACCGGTAA
Me7: TGAGTCCAAACCGGTCG	Me8: TGAGTCCAAACCGGTGC	Me9: TGAGTCCAAACCGGTCA
Me10: TGGGGACAACCCGGCTT	Me11: TGAGTCCAAACCGGTGT	Me12: GGTGAACGCTCCGGAAG
Me13: AGCGAGCAAGCCGGTGG	Me14: TGAGTCCAAACCGGTTG	Me15: TGAGTCCAAACCGGTAG
Me16: GAGTATCAACCCGGATT	Me17: TGAGTCCAAACCGGGCT	Me18: TACGACGAATCCGGACT
Me19: CACAGTCATGCCGGAAT	Me20: CTTACTTAGACCGGAGT	Me21: TGAGTCCAAACCGGACA
**Reverse primers and sequence (5’-3’)**		
Em1: GACTGCGTACGAATTAAT	Em2: GACTGCGTACGAATTTGC	Em3: GACTGCGTACGAATTGAC
Em4: GACTGCGTACGAATTTGA	Em5: GACTGCGTACGAATTAAC	Em6: GACTGCGTACGAATTGCA
Em7: GACTGCGTACGAATTCAA	Em8: GACTGCGTACGAATTCTG	Em9: GACTGCGTACGAATTCGA
Em10: GACTGCGTACGAATTCAG	Em11: GACTGCGTACGAATTCCA	Em12: GACTGCGTACGAATTGTC
Em13: GACTGCGTACGAATTGGT	Em14: GACTGCGTACGAATTCGG	Em15: GACTGCGTACGAATTATG
Em16: GACTGCGTACGAATTAGC	Em17: AGGCGGTTGTCAATTGAC	Em18: GACTGCGTACGAATTACG
Em19: GACTGCGTACGAATTATT	Em20: GACTGCGTACGAATTTAG	Em21: GACTGCGTACGAATTTCG

### Capillary electrophoresis detection

For increased efficiency and accuracy of genotyping, the mapping population was genotyped using polymorphic primer combinations labeled with 5’-HEX under PCR conditions ibidem, and the PCR products were sent to Sangon Biotech (Shanghai) Co., Ltd (Shanghai, China) for capillary electrophoresis according to the methods described in literatures [39, 40]. Briefly, 50 pg of amplified product was added to a mixture containing 990 μL Hi-Di formamide (Applied Biosystems) and 10 μL internal lane standard (GS1200LIZ, Applied Biosystems) after quantification, and the bands were separated using a DNA Analyzer (3730xl, Applied Biosystems) with 50-cm capillaries filled with POP-7 separation matrix (Applied Biosystems) [39]. Capillary array system from Applied Biosystems (Foster City, CA, USA) is one of the most commonly used systems for sequencing and fragment analysis [41]. Data were collected using Data Collection software (version 4.0, Applied Biosystems) and 150 FSA file were obtained for the mapping population with 148 DH and two parents after amplified using one of the primer combinations.

### Fragment analysis and genotyping

The corresponding 150 FSA files were then analyzed using GeneMarker program (version 2.7.1, Softgenetics, LLC) under the analysis type ‘Fragment (plant)’ [41, 42]. The peak detection threshold was set at 200 RFU and the fragment size was set to be 100–1,000 bp. GS1200LIZ size standard was used as an internal lane size standard which enabled automated data analysis, and was also essential for achieving high run-to-run precision in sizing DNA fragments [43]. An Excel document including the information of amplified bands (e.g. size and peak height) was exported after each analysis when report style ‘Bin Table (AFLP/MLPA)’ was selected. During fragment analyzing, size calling of some samples was failed probably because of PCR or electrophoresis failure. The genotypes of these samples were used as missing data in linkage analysis. In this study, if one primer combination missed data of more than four DH strains or of one parental strain, the data of the primer combination would not be used for map construction.

### Construction of genetic linkage map

The amplified products of every primer combination were analyzed and only loci which were polymorphic between the two parents and were segregated among the 148 DH population were selected for linkage analysis using the JoinMap program (version 4.0, Kyazma B.V.) [44]. Loci data were first transformed into the formats of JoinMap. Briefly, loci identical to the maternal parent Py-HT were manually recorded as ‘a’, those identical to the paternal parent Py-LS were recorded as ‘b’, and the missing loci were recorded as ‘-’. Population type was selected as ‘DH1’. Genotype frequency of each locus was calculated and the loci with too many missing data were excluded. Chi-square (χ^2^) test was performed to determine whether the genotypic frequency at each locus was deviated from the expected 1:1 segregation ratio. Normal segregation was considered as *P* ≥ 0.01 and low-level skewed segregation was considered as 0.001 ≤ *P* < 0.01. Severely skewed segregation loci with *P* < 0.001 were not used for linkage analysis.

Constructing a genetic linkage map involves a stepwise approach [45, 46]. First, markers with *P* ≥ 0.001 were divided into different linkage groups (LGs) using command ‘Create groups using the grouping tree’. The markers in each group were ordered under the major criteria of a maximum recombination fraction of 0.4 and a minimum LOD score of 1.0 using ‘Regression Mapping’ method [47]. The distance between the markers was calculated using Haldane’s mapping function [48]. QTL IciMapping program (version 4.1, CAAS) was used to output the graphical presentation of the genetic linkage map [49].

SRAP loci were labelled according to the primer combination employed and their estimated fragment length, e.g. ‘M19E9-180.6’ designated a locus that yielded a 180.6 bp fragment with the primer combination of Me19 and Em9 (Table 1). The name of a skewed marker was suffixed by ‘D’, for example, ‘M13E2-473.6D’. Therefore, the positions of distorted markers can be easily observed from the genetic linkage map. If the markers were clustered in special regions on chromosomes, these regions were designated as segregation distortion regions (SDRs) [50–52]. The presence of a SDR was declared when two or more distorted markers were clustered. The direction of distortion was determined by comparing the information of each locus with parental genotypes.

### Genome length and map coverage

The expected size of *P*. *yezoensis* genome (*L*) was estimated using two different methods. In one method, *L*_1_ was calculated as the summed length of all LGs plus two times of the average marker spacing [53]. In the other method, *L*_2_ was calculated as the length of each LG multiplied by the factor (m+1)/(m-1), where m is the number of markers on each LG [54]. The estimated *L* was the average of the lengths calculated by the above two methods. Map coverage was estimated by the ratio between the cumulative map length and the expected genome size.

### Marker distribution

Marker distribution along each LG was evaluated by comparing the difference between the expected positions of the markers and the observed ones using Kolmogorov-Smirnov test (α = 5%) as described by Lombard and Delourme [55]. Online package KS-test was used to calculate the corresponding *D* value and *P* value of the test [56]. A random distribution of markers on each LG was indicated when *D* < *D* (Ni, 0.05) or *P* > 0.05. The values of *D* (Ni, 0.05) were described by Jerrold [57].

### QTL mapping

Phenotypic values of six economic traits of F_1_ gametophytic blades of the mapping population were determined in our previous work [30] and shown in S1 Table. Briefly, conchospores released from mature conchocelis of each DH strain were collected and cultured into blades [58]. The length, width and fresh weight of the blades on the 40th and 50th day were determined (L40, L50, W40, W50, FW40 and FW50). The blade length was measured from the holdfast to the blade tip. The blade width was measured at the widest part of the blade. The fresh weight of the blade was measured after the surface water of the blade was sucked up with a paper towel. The specific growth rate of blade length, width and fresh weight between the 40th and 50th day (LGR, WGR and FWGR) were calculated based on a formula. Take LGR for example, $\mathrm{LGR}\;(\%)=\frac{\ln\left(L50\right)‑\ln(L40)}{n}\times 100$, where ln was the natural logarithm, L50 and L40 were the length of blade at 40th and 50th day, respectively, and n was the interval of 10 days (http://dx.doi.org/10.17504/protocols.io.x3vfqn6). QTL was analyzed with QTL IciMapping program (version 4.1, CAAS) using the ICIM-ADD method (inclusive composite interval mapping of additive and dominant QTL) [49, 59, 60]. A stringent LOD threshold 1.5 was set to identify the putative presence of QTLs associated with economic traits of blades. A QTL was declared when the LOD value was higher than the threshold of 1.5 [61]. QTLs were named and shown in italic by prepending a lower-case ‘q’ to the abbreviation of a trait name, followed by the serial number of LGs where the QTL was found, and a terminal number providing a unique number to distinguish multiple QTLs of one trait on a single chromosome [62, 63], e.g. ‘*qL50-2-1*’ designated the first QTL of L50 detected on LG2.

## Results

### Genotyping

After screening, four forward primers (Me4, Me7, Me13 and Me19) and three reverse primers (Em6, Em8 and Em10) were labeled with 5’-HEX and paired with ordinary primers to analyze the mapping population. Genotyping by means of capillary electrophoresis directly provided the digitized information of the fragments (Table 2), which was more efficient than PAGE method [64].

**Table 2:** Partial digitized information of sequence-related amplified polymorphism fragments of six samples analyzed using GeneMarker software based on the results of capillary electrophoresis.

Sample name	Peak height (RFU) of amplified fragment with different size (bp)							
	106.4	107.4	139.9	140.8	175.9	176.9	188.0	189.0
**108-HT**	-	13500	-	-	-	-	3132	2424
**108-LS**	4199	4849	-	-	2137	3167	2365	2759
**108–031**	8630	-	1782	1860	-	-	1171	-
**108–032**	-	-	-	-	-	-	-	-
**108–033**	-	18946	38962	24087	1020	-	-	-
**108–034**	-	-	16119	10153	-	-	-	-

‘-’ denoted that no fragment with peak height ≥ 200 RFU was amplified.

Data of 79 primer combinations with missing data number less than or equal to four DH strains per primer combination were used for genetic mapping. As shown in S2 Table, a total of 42,049 loci were amplified, of which 5,661 loci were amplified in parents with 5,172 polymorphic loci (91.36%). In addition, 5,059 loci were amplified in both parents and the mapping population, of which 4,570 (90.33%) were polymorphic between the parents and were segregated among 148 DH population.

### Map construction

For map construction, 4,570 SRAP loci that met the requirement of linkage analysis were imported into JoinMap and evaluated with χ^2^ test. We found that 301 loci were segregated with expected 1:1 ratio at *P* ≥ 0.01, and 96 loci were low-level skewed at *P* values 0.001~0.01 (S3 Table). Meanwhile, 3,775 loci with serious segregation distortion (*P* < 0.001) were discarded. Ultimately, a total of 397 loci including 227 Py-HT-specific loci and 170 Py-LS-specific loci were used for linkage analysis (S3 Table). Approximately, each primer pair amplified five informative loci.

**Fig 1:**
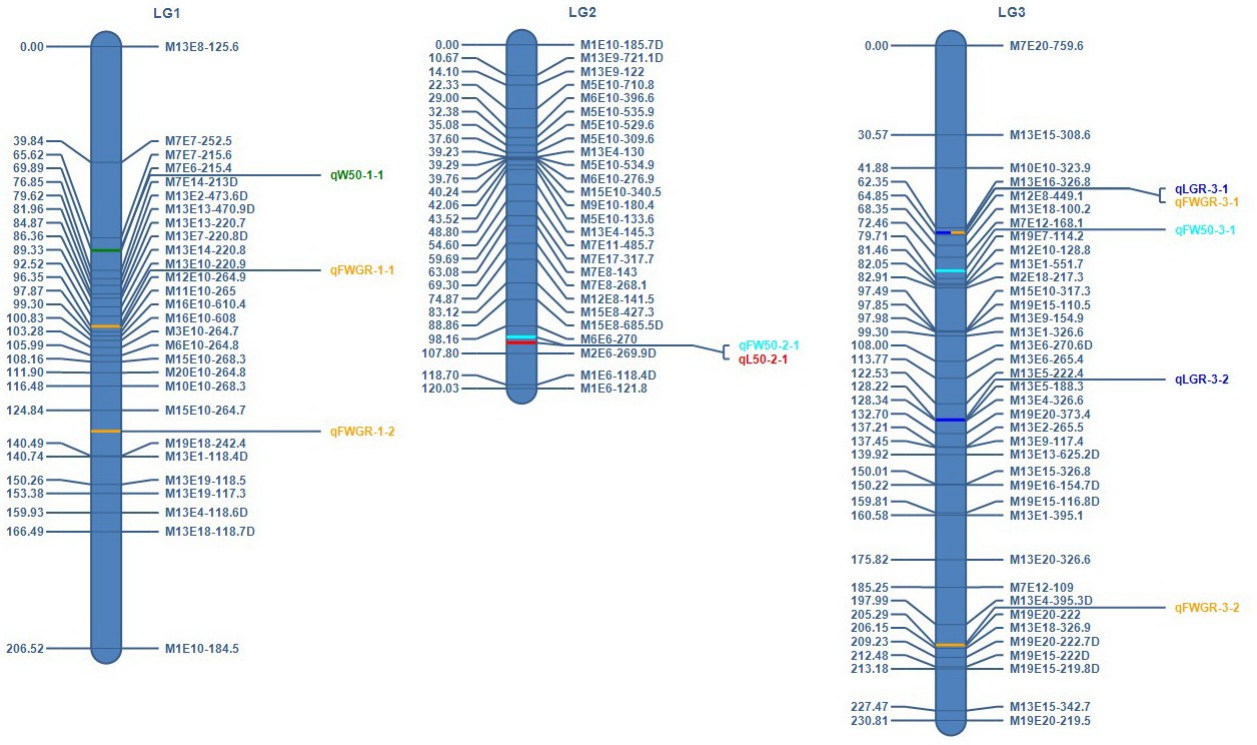
Distribution of quantitative trait loci (QTLs) controlling five economic traits of gametophytic blades on a genetic linkage map of *Pyropia yezoensis* constructed with sequence-related amplified polymorphism markers based on a doubled haploid population. The designations on the right are marker names, on the left are mapped distances in centimorgans based on Haldane’s mapping function. The colored bars denote QTLs positions and the names of QTLs are next to the long lines on the right. Loci showing low-level segregation distortion (0.001 ≤ *P* < 0.01) are indicated with a letter ‘D’ suffix.

At LOD 7.0, two groups containing 118 and 94 markers, respectively, were used to construct LG1 and LG2, respectively. The remaining markers were moved to a new group and those with LOD values above 4.0 were used to construct LG3. Finally, the genetic linkage map of *P*. *yezoensis* was constructed using the three LGs (LG1-LG3) (Fig 1). The map included 92 SRAP markers and spanned a total distance of 557.36 cM, with a mean interlocus space of 6.23 cM between adjacent markers (Table 3). The number of LGs in the genetic linkage map was equal to the chromosome number of the haploid genome (3 chromosomes) of *P*. *yezoensis* [65].

**Table 3:** Information on the genetic linkage map of *Pyropia yezoensis* constructed with sequence-related amplified polymorphism markers based on a doubled haploid population.

ID	Number of markers	Length (cM)	Interlocus space (cM)			Number of gaps > 20 cM
			Mean	Min.	Max.	
**LG1**	28 (7)	206.52	7.65	0.25	40.03	3
**LG2**	26 (5)	120.03	4.80	0.06	10.90	0
**LG3**	38 (8)	230.81	6.24	0.12	30.57	2

Numbers in parentheses denote the number of low-level segregation distortion markers.

Maximum marker spacing on the three LGs were between M13E18-118.7D and M1E10-184.5 (40.03 cM), M2E6-269.9D and M1E6-118.4D (10.90 cM), M7E20-759.6 and M13E15-308.6 (30.57 cM), respectively. The minimum spacing on the three LGs were between M19E18-242.4 and M13E1-118.4D (0.25 cM), M13E4-130 and M6E10-534.9 (0.06 cM), M13E5-188.3 and M13E4-326.6 (0.12 cM), respectively. Furthermore, there were five gaps large than 20.0 cM, including three gaps larger than 30.0 cM and one gap larger than 40.0 cM. On a SNP-based linkage map of *L*. *japonica*, the largest gap was 14.97 cM although the mean interlocus space was 0.36 cM [27]. Those large gaps might be due to the lack of enough polymorphisms in some chromosome regions between mapping parents [66]. Large numbers of additional genetic markers [67, 68] or mapping population from different cross combinations [69] are needed to fill in the gaps to provide a higher-resolution map.

### Genome coverage and marker distribution

Based on the total data set of the mapping population, the average estimate of expected genome length was 597.74 cM. Thus, the map covered 93.71% of the estimated genome of *P*. *yezoensis*. The results of Kolmogorov-Smirnov test showed that *D* values of LG1, LG2 and LG3 were 0.21, 0.19 and 0.18, respectively, which were all smaller than the corresponding values of *D* (Ni, 0.05) (0.25, 0.26 and 0.22, respectively). The corresponding *P* values of the three LGs were 0.49, 0.67 and 0.50, respectively, which were all larger than 0.05. These results indicated that the markers along the three LGs were uniformly distributed, which was an important feature of a high quality genetic linkage map [55].

### Segregation distortion

There were six SDRs containing 2–3 clustered distorted markers with the same skew directions after compared with the genotypes of the parent strains. The loci within the SDRs on LG1 and LG2 skewed toward Py-HT and Py-LS, respectively, and the loci within the first and second SDR on LG3 skewed toward Py-LS and Py-HT, respectively (Table 4). Besides, there were six isolated segregation distortion loci. Among these loci, M13E7-220.8D, M13E1-118.4D, M13E13-625.2D and M13E4-395.3D skewed toward Py-HT; and M15E8-685.5D and M13E6-270.6D skewed toward Py-LS. These regions may contain segregation distortion related genes [70, 71]. Further study is needed to explore it.

**Table 4:** Information of segregation distortion regions (SDRs) on the genetic linkage map of *Pyropia yezoensis*.

ID	Location	Segregation distortion marker	Skew direction
**SDR1**	LG1	M13E13-470.9D, M7E14-213D, M13E2-473.6D	Py-HT
**SDR2**	LG1	M13E18-118.7D, M13E4-118.6D	Py-HT
**SDR3**	LG2	M1E10-185.7D, M13E9-721.1D	Py-LS
**SDR4**	LG2	M2E6-269.9D, M1E6-118.4D	Py-LS
**SDR5**	LG3	M19E15-116.8D, M19E16-154.7D	Py-LS
**SDR6**	LG3	M19E15-222D, M19E20-222.7D, M19E15-219.8D	Py-HT

### QTL mapping

In general, marker distance should be less than 10.0 cM for a map used for QTL analysis [72–74]. In *L*. *japonica*, the marker distance was 6.7 cM on the first map with a coverage of 82.8% for QTL mapping of frond length and width [26].The present map covered 93.71% of the genome and the SRAP markers along each LG were evenly distributed with an average distance of 6.23 cM between adjacent markers, indicating that the map can be used for QTL detection of blade traits in *P*. *yezoensis*. In total, 10 QTLs associated with L50, W50, FW50, LGR and FWGR were identified (Fig 1 and Table 5). However, WGR associated QTL was not detected. One QTL for L50 was found on LG2 and one QTL for W50 was identified on LG1, with phenotypic variance explained (PVE) of 5.72 and 7.05%, respectively. Two QTLs for FW50 were identified on LG2 and LG3, with PVE of 4.84 and 6.45%, respectively. Two QTLs for LGR were on LG3, with PVE of 7.87 and 3.23%. Four QTLs for FWGR were on LG1 and LG3 with PVE ranging 2.29–4.53%.

**Table 5:** Quantitative trait loci (QTLs) of five economic traits of gametophytic blades of *Pyropia yezoensis*.

QTL	Position (cM)	Left marker	Right marker	LOD values	PVE (%)	Add values	Interval of confidence (cM)	
							Left	Right
***qW50-1-1***	70.00	M7E6-215.4	M7E14-213D	1.7	7.05	0.04	67.50	74.50
***qFWGR-1-1***	96.00	M13E10-220.9	M12E10-264.9	1.8	2.35	1.16	92.50	97.50
***qFWGR-1-2***	132.00	M15E10-264.7	M19E18-242.4	1.7	3.20	1.35	125.50	140.50
***qFW50-2-1***	102.00	M6E6-270	M2E6-269.9D	2.3	4.84	2.49	87.50	107.50
***qL50-2-1***	104.00	M6E6-270	M2E6-269.9D	2.0	5.72	0.83	91.50	107.50
***qLGR-3-1***	64.00	M13E16-326.8	M12E8-449.1	2.1	7.87	0.90	63.50	66.50
***qFWGR-3-1***	64.00	M13E16-326.8	M12E8-449.1	1.6	4.53	1.61	62.50	66.50
***qFW50-3-1***	77.00	M7E12-168.1	M19E7-114.2	1.9	6.45	-2.83	73.50	80.50
***qLGR-3-2***	128.00	M13E5-222.4	M13E5-188.3	1.6	3.23	0.59	122.50	128.50
***qFWGR-3-2***	205.00	M13E4-395.3D	M19E20-222	1.7	2.29	-1.17	201.50	205.50

PVE (%) denotes phenotypic variation explained by a QTL. Add denotes estimated additive effect of a QTL.

According to literatures, PVE of major QTLs should be larger than 15% [75–77], or at least larger than 10% [78]. PVE of QTLs in the present study was 2.29–7.87%, indicating that all of the QTLs had minor effects. Generally, only loci with major effects would undergo map-based cloning and be thoroughly studied [79]. In recent years, more attention has been paid to QTLs with relatively minor effects and studies have shown that minor-effect QTLs also make important contributions [80]. However, they may have inconsistent additive effects under different genetic backgrounds and environments and selection of reliable candidates for further study remains a challenge [80].

LOD values of the 10 QTLs ranged 1.6–2.3, and three of them were ≥ 2.0 (Table 5). Although it is not clear what should be the minimum LOD value for declaring a significant QTL [81], LOD scores of 2.0–3.0 are commonly used to control the probability of overall false positives within 0.05 [25, 82, 83]. Higher LOD values could better control the occurrence of false QTLs and is suitable for fine mapping of major QTLs [84, 85]. However, true QTLs with minor genetic effects are hard to detect at high LOD values, suggesting that LOD threshold should be reduced if more minor QTLs need to be detected for marker-assisted breeding [83]. Therefore, the QTLs with different LOD values detected in the current study could be used for different researches.

Eight of the 10 QTLs had positive values of additive effect (Table 5), indicating that their favorable alleles originate from the maternal parent. The remaining two QTLs had negative Add values, indicating that their favorable alleles originate from the paternal parent [86, 87]. Our result was in accordance with the fact that most characters of maternal parent Py-HT were superior to paternal parent Py-LS [32]. Additive effect occurs when two or more genes source a single contribution to the final phenotype [88].

The interval of confidence (IC) was 3.0–7.0 cM in seven QTLs and 15.0–20.0 cM in the other three QTLs (Table 5). The higher IC for a QTL, the more genes may be involved. In this circumstance, it is difficult to determine whether the QTL is composed of a single gene with large effect or multiple genes with smaller effect. ICs of 15.0–20.0 cM was too high for position cloning [89] and should be narrowed down for the precise estimate of QTL position [90, 91]. The Complex Trait Consortium considered that IC should be less than 1.0–5.0 cM for fine mapping [92]. Therefore, four QTLs (*qFWGR-1-1*, *qLGR-3-1*, *qFWGR-3-1* and *qFWGR-3-2*) in the present study met the requirement and could be used for further study.

Two clusters of QTLs were detected on two of the three LGs (Fig 1). The first cluster contained two QTLs (*qL50-2-1* and *qFW50-2-1*) that were located on LG2 within the 98.16–107.80 cM region and were 2.00 cM apart from each other. The distance between *qL50-2-1* and the nearest marker M2E6-269.9D was 3.80 cM and between *qFW50-2-1* and its nearest marker M6E6-270 was 3.84 cM. The IC of *qFW50-2-1* covered the IC of *qL50-2-1*. The second cluster contained two QTLs (*qLGR-3-1* and *qFWGR-3-1*) located on LG3 within the 62.35–64.85 cM region at the same position. The distance between these two QTLs and the nearest marker M12E8-449.1 was 0.85 cM. The IC of *qFWGR-3-1* covered the IC of *qLGR-3-1*. It is considered that a marker should co-segregate or be closely linked to the desired trait, and the distance between a QTL and the nearest marker ≤ 1.0 cM can be a threshold for MAS [93]. Therefore, six QTLs in the present study with a distance of less than 1.0 cM from the nearest markers could be used for further study.

## Discussion

Historically, color mutants are used as genetic markers for crossbreeding and genetic study of *P*. *yezoensis* [9, 29]. The distances between the centromeres and the loci of four color mutants have been determined and assigned to three different LGs [65], which could be considered as a traditional genetic linkage map. However, the number of markers contained in this map is too small and thus the information provided by this map is far from that needed for further study. In the present study, for the first time, we constructed a genetic linkage map of *P*. *yezoensis* that contained 92 polymorphic SRAP markers and 10 QTLs associated with economic characters of blades based on a DH population. The map may provide a reference for molecular breeding in *P*. *yezoensis*.

Mapping population is critical for linkage analysis and is usually obtained from controlled cross between the crossing parents that have sufficient variation for traits of interest at both DNA and phenotypic levels [94]. Theoretically, the higher the variation, the easier to obtain abundant recombination. However, the parents should not be so diverse that they are unable to cross [95]. Our previous study demonstrated that the crossing parents (Py-HT and Py-LS) had significant differences in blade traits and several recombinant strains had been screened [32]. Besides, in our previous work, we found that the genetic similarity index between the two parents was 0.4962 [96], which suggested a high genetic diversity between them. In the present study, 91.36% polymorphic loci were found between the parents after SRAP analysis. Therefore, DH population constructed based on the cross of Py-HT × Py-LS [30] was used for the genetic mapping. A DH is a genotype formed when a haploid cell undergo induced or spontaneous chromosome doubling [97]. DH can be exploited to produce completely homozygous lines, construct genetic linkage maps, locate genes of economic importance and increase breeding efficiency [98]. DH is especially powerful for analyzing quantitative traits because replicated traits can be analyzed re-using identical genetic material [99].

In *P*. *yezoensis*, the blades are monoecious and could be self-fertilized. Therefore, the DH population can be established by self-fertilization because spermatia (male gamete) and carpogonium (female gamete) always occur diffusely on a single sector [65, 100, 101] that is developed from one of the tetrad cells after mitosis [65]. Thus, the gametes formed on a single color-sector are genetically identical. We considered that self-fertilization of a color-sector was a procedure of chromosome doubling of gamete. DH population of 148 strains was constructed using 37 four-color sectored F_1_ gametophytic blades [30]. Every strain of the DH population was obtained from one single color-sector and developed from one carpospore after self-fertilization of the sector. Therefore, a DH of *P*. *yezoensis* is similar to a DH of higher plant in that both of them are homozygous diploid.

Based on simulation studies, the type and size of experimental population can exert an influence on the accuracy of a genetic linkage map [102]. The higher the number of individuals, the more precise is the map, but at the same time larger population means excessive work and costs associated with phenotyping and genotyping [103, 104]. It is important to select a population with appropriate size. Most experiments have used a total of 100 to 200 individuals or progenies [25, 55, 105]. In the present study, we used 148 DH strains, which was in similar size (157 strains) to *P*. *haitanensis* in a similar study [23].

Molecular markers are important tools for creating a genetic linkage map and have significantly increased the genetic knowledge in many cultivated plant species [14]. SRAP markers used in the present study is a PCR marker system that combines simplicity, reliability and a moderate throughput ratio [37]. It has been extensively used in genetic diversity analysis [106, 107] and genetic mapping in economic plants [14, 37, 108, 109], including *P*. *haitanensis*, one of the most important seaweeds in China, when there are not enough SSR markers [23]. The present map can be further saturated with SSR markers that could be developed from genome sequence of *P*. *yezoensis* [28]. SRAP can also be useful for QTL mapping because of their ability to target gene-rich regions of the genome [14]. The quantitative trait data can be used to determine if any SRAP markers are closely associated with those traits [110]. Once the markers are identified, breeders can select desirable QTLs without interference from environmental effects [111].

During the construction of a molecular genetic linkage map, the most difficult and complicated steps are the separation of PCR products and detection of polymorphic bands. The traditional method used to separate PCR products and detect polymorphic bands is PAGE [37]. However, PAGE cannot give the accurate size of DNA fragments and its detection efficiency is low. Besides, PAGE may have some degree of error when the results were manually recorded. Our previous study found that 11 SRAP primer combinations amplified 95.42% polymorphic bands in six strains of *P*. *yezoensis*, with an average of 11.4 loci per primer combination [96]. The abundant polymorphism met the requirement of a molecular marker for genetic mapping, but in the meantime added difficulties to the artificial recording of bands. To solve this problem, we performed capillary electrophoresis with fluorescence detection in the present study, which has advantages of high separation efficiency, short analysis time and high-throughput [112].

During genetic mapping, a phenomenon called segregation distortion, which means that many markers deviate from the expected Mendelian fraction, is often encountered [19, 113]. Segregation distortion has been found in many plant studies and is considered one of the main evolutionary forces [114–116]. Various factors have been suggested to cause segregation distortion [117]. However, the underlying mechanism is still debated and obscure [118–120]. For DH population, high percentage of segregation distortion may be caused by strong zygotic selection, which refers to the gametophytic competition during zygote formation [119, 121]. The percentage of skewed SRAP markers (24.18%) of the DH population in the present study was less than that (30.10%) reported previously in *P*. *haitanensis* [23]. Several studies show that segregation distortion affects the estimation of genetic distance and the order of markers on the same LG [122, 123]. Skewed markers may have some genetic information, but their accuracies are unknown. Thus, some researchers think they should be ignored to obtain more accurate genetic linkage maps [124], as in the study of *P*. *haitanensis* [23]. However, if distorted markers are ignored, map coverage may decrease and some important information in the real data analysis of QTL mapping may lose [125, 126]. Several genetic linkage maps established using second-generation markers contain some skewed markers [120, 127, 128]. In the present study, the genetic linkage map contained 20 skewed markers, of which 14 markers formed six SDRs, and the map coverage was 93.71% of the genome, which was higher than that (88.1%) of a genetic map in *P*. *haitanensis* without skewed markers [23].

Theoretically, the number of LGs should be consistent with the number of haploid chromosomes of a species, as in the study of *P*. *haitanensis* [23]. This is because two homologous chromosomes possess either similar or allelic genes on the same loci, which constitute the same LG [129, 130]. However, we did not find a LOD value that could divide all the markers into three groups. Based on the literature, different LOD values can be applied to different LGs of a species in specific situations [130]. Thus, the LOD values of the three LGs on the constructed map were 7.0, 7.0 and 4.0 in the present study.

We found two clusters composed of QTLs for different traits on LG2 and LG3. The phenomenon of QTL cluster exists widely in crops [118, 131] and was also found in *P*. *haitanensis* [25]. Traits clustered within the same region are significantly correlated with each other [30, 132]. This cluster phenomenon could be considered as multifactorial linkages followed by natural selection favoring co-adapted traits, which is partly due to pleiotropy of some unknown key factor(s) controlling various traits through diverse metabolic pathways [133].

## Conclusions

The SRAP genetic linkage map constructed in the present study provided a framework for linkage analysis and QTL detection in *P*. *yezoensis*. By saturating the map and validating these QTLs, functional markers could be identified or converted for future marker-assisted breeding.

## Supporting information

S1 Table Information on the genetic linkage map and economic traits data for QTL mapping. Sheet 1 is the marker information on the genetic linkage map, Sheet 2 is the loci information used to construction the genetic linkage map, Sheet 3 is the six economic traits data of the DH mapping population.
(XLSX)

S2 Table Amplified loci in parents and doubled haploid (DH) population of *Pyropia yezoensis* analyzed by fluorescent sequence-related amplified polymorphism markers. (XLSX)

S3 Table Segregation of sequence-related amplified polymorphism loci in the doubled haploid population of *Pyropia yezoensis*. (XLSX)
